# Cytotoxicity of Doxycycline Effluent Generated by the Fenton Process

**DOI:** 10.1155/2014/439461

**Published:** 2014-10-14

**Authors:** Alexandre Augusto Borghi, Laura Oliveira-Nascimento, Marco Antônio Stephano, Paula Monteiro de Souza, Attilio Converti, Mauri Sérgio Alves Palma

**Affiliations:** ^1^Department of Biochemical and Pharmaceutical Technology, Faculty of Pharmaceutical Sciences, University of São Paulo, Bloco 16, Avenida Prof. Lineu Prestes 580, 05508-900 São Paulo, SP, Brazil; ^2^Department of Biochemistry and Tissue Biology, Institute of Biology, University of Campinas, University Town Zeferino Vaz, Rua Monteiro Lobato 255, 13083-862 Campinas, SP, Brazil; ^3^Department of Civil, Chemical and Environmental Engineering, Pole of Chemical Engineering, Genoa University, Via Opera Pia 15, 16145 Genoa, Italy

## Abstract

This study aims at determining the Minimum Inhibitory Concentration with *Escherichia coli* ATCC 25922 and cytotoxicity to L929 cells (ATCC CCL-1) of the waste generated by doxycycline degradation by the Fenton process. This process has shown promise in this treatment thanks mainly to the fact that the waste did not show any relevant inhibitory effect on the test organism and no cytotoxicity to L-929 cells, thus demonstrating that the antibiotic properties were inactivated.

## 1. Introduction 

The growing antibiotics consumption by humans and animals has increased their concentrations in wastewaters either as such or in the form of metabolic products. Since the conventional treatments are unable to degrade their molecules, they are usually released directly into the receiving water bodies. As a result, the release of these compounds into the environment has led to the selection of resistant pathogens, genetically able to transmit such a characteristic to their offspring.

Ghosh et al., studying the action of five antibiotics (clarithromycin, enrofloxacin, sulfamethoxazole, tetracycline, and trimethoprim) present in four different effluents on bacteria responsible for ammonia oxidation in sewage treatment plants, observed no significant individual effect of these drugs at concentrations below 0.05 mg/L [1].

Jia et al., using a mass detector coupled to liquid chromatograph, detected the presence of oxytetracycline, tetracycline, and their degradation products (4-epi-tetracycline, 4-epi-oxytetracycline, isochlortetracycline, anhydrotetracycline, and 4-epi-anhydrotetracycline) at concentrations in the range 1.9–72.5 ng/L in the influent and effluent from the sewage treatment plant in Beijing (China), and even in surface water (2.1-2.2 ng/L) [2].

The presence of tetracyclines in wastewaters from various sources has spurred studies of new oxidation methods for their treatment, with particular attention to the degradation mechanisms and the identification of degradation products, most of which are based on the generation of hydroxyl radicals [3].

Gujarathi et al., who used phytoremediation to treat effluents containing tetracycline and oxytetracycline, observed that the molecules of these antibiotics were almost completely degraded within 6 and 15 days by radicals and enzymes present in the roots of* Myriophyllum aquaticum* and* Pistia stratiotes*, respectively, at rates decreasing with the increase in their initial concentrations [4].

Jiao et al. studied the photocatalytic degradation of tetracycline and the toxicity of its degradation products with mass/charge between 398 and 413 [5]. Naphthol moiety of tetracycline remained intact during photolysis, while the toxicity of degradation products to* Vibrio fischeri *as target microorganism increased with the irradiation time.

Yuan et al. successfully used hydrogen peroxide combined with UV radiation to degrade oxytetracycline, doxycycline, or ciprofloxacin at a concentration of 5 *μ*M in water samples of different origins (ultrapure water, surface water, drinking water, and effluents from a municipal sewage treatment plant) [6]. The degradation products from the UV treatment showed toxicity against* V. fischeri* similar to that of antibiotics, while those from the combined UV/H_2_O_2_ treatment, after an initial phase of increased toxicity, exhibited no toxicity.

Mboula et al., who investigated tetracycline degradation by a heterogeneous photocatalytic process consisting in the combined use of TiO_2_ and UV [7], observed a 24% reduction in the concentration of dissolved organic carbon, a decrease in toxicity against* Pseudomonas aeruginosa*, and nonbiodegradability of the degradation products according to the Sturm test [8, 9]. HPLC-ESI (+)-MS/MS analysis of products showed that the tetracycline ring was preserved, thus proving that their structures were not so different from that of the starting compound.

Tetracyclines are the third most consumed antibiotics, after penicillins and quinolones, and, due to their indiscriminate use, an increasing number of bacteria resistant to tetracyclines have been detected [10]. Among them, doxycycline is widely used for the treatment of infectious diseases caused by rickettsiae, chlamydiae, and mycoplasmas [11]. However, new applications for this drug were discovered and have contributed to their increased use in recent years, among which are the treatment of anthrax, lyme disease, malaria, inflammation, and pneumonia caused by methicillin-resistant* Staphylococcus aureus* strains [12, 13]. Because of its wide use, it is present in industrial and domestic effluents as well as those from rural areas in concentrations up to 6.7 mg/L [14]. Thus, it becomes necessary to set up efficient and cheap techniques to treat these effluents.

Hydrogen peroxide is an excellent oxidant, whose oxidant power is exploited in the Fenton process, in the presence of ferrous ion as a catalyst and in the absence of light, to form the hydroxyl radical (OH^•^) that is capable of destroying most organic pollutants [15–17].

Based on this background, the scope of this study was to determine the toxicity of products generated by doxycycline degradation through the Fenton process to establish whether it could be successfully used in the treatment of effluents containing this antibiotic. To achieve this goal we determined in this study the minimum inhibitory concentration and cytotoxicity of laboratory-produced samples containing degradation products of such an antibiotic using* Escherichia coli* ATCC 25922 and L929 cells (ATCC CCL-1), respectively.

## 2. Materials and Methods

### 2.1. Materials

Sulfuric acid, 33% hydrogen peroxide, sodium hydroxide, and ferrous sulfate heptahydrate were purchased from Synth (Diadema, São Paulo, Brazil), barium chloride and sulfate from Merck (Darmstadt, Germany), 91.9% doxycycline hydrochloride from Calbiochem (San Diego, CA, USA), Luria Bertani (LB) medium from Difco Laboratories (Detroit, MI, USA), and 3-(4,5-dimethylthiazol-2-yl)-2,5-diphenyltetrazolium bromide (MTT), 0.4% Tripan Blue, 0.25% trypsin EDTA, and Dubecco's Modified Eagle Medium (DMEM) low glucose from Sigma-Aldrich (St. Luis, MO, USA).

All chemical compounds, except the solutions, were high purity grade reagents.

### 2.2. Toxicity Tests

For all the toxicity, minimum inhibitory concentration (MIC) and cytotoxicity tests, a stock solution of 2000 mg/L doxycycline was prepared by diluting 0.1 g of the antibiotic in a 50 mL-graduated flask with type I purified water previously sterilized by filtration through membranes with 0.22 *μ*m-pore diameter.

Toxicity tests were carried out at 37°C in a 300 mL-beaker protected from light containing 200 mL of the stock doxycycline solution diluted to 100 mg/L, 25 mg/L Fe^2+^, and 611 mg/L H_2_O_2_. After 240 min of reaction, 0.1 M NaOH was added to a 20 mL aliquot of this solution up to pH 13 to precipitate Fe^3+^; the resulting suspension was filtered through a membrane with 0.45 *μ*m-pore diameter and the pH of the filtrate adjusted to 7.0 with 1 : 5 (v/v) H_2_SO_4_. The negative control, simply containing 9 mg/L H_2_O_2_ and 25 mg/L Fe^2+^, was prepared and let to react exactly in the same way.

All solutions were sterilized by filtration through membranes with 0.22 *μ*m-pore diameter in a laminar flow chamber prior to their use in tests.

### 2.3. Determination of the Minimum Inhibitory Concentration

The minimum inhibitory concentration (MIC) was determined in microplates following the agar dilution method according to the protocol M07-A8 of the Clinical and Laboratory Standards Institute and National Committee for Clinical Laboratory Standards [18].* Escherichia coli* ATCC 25922 was used as the control strain.

Petri dishes containing the solid and liquid LB media were prepared according to the manufacturer using deionized water and then sterilized in autoclave at 120°C for 25 min and finally incubated at 37°C for 24 h to check the effectiveness of sterilization.

The antibiotic solution for this determination was obtained by diluting the stock solution with sterilized type I purified water up to a concentration of 1000 mg/L.

To standardize the inoculum density, we used a BaSO_4_ turbidity standard equivalent to a 0.5 McFarland standard, that is, ~1 to 2*·*10^8^ colony forming units (CFU)/mL. The standard solution was prepared by diluting 0.5 mL of 0.048 mol/L BaCl_2_ with 99.5 mL of 0.18 mol/L H_2_SO_4_ under stirring and its optical density at 625 nm determined by an UV-Vis spectrophotometer, model DU 640 (Beckman, Orange County, CA, USA).

Initially, a loopful of cells was inoculated in petri dishes containing LB agar medium and the dishes were incubated at 37°C for 18–24 h. Single colonies were selected and resuspended in 5 mL of 0.9% (w/v) sterile saline. Turbidity was measured by UV-Vis spectrophotometry at 625 nm after adding every colony and the addition was stopped when the optical density of the bacterial suspension reached the value of the standard solution.

The susceptibility of the control strain to doxycycline was determined by the microdilution assay. For this purpose, the stock antibiotic solution was firstly diluted in a 96-well microplate (100 *μ*L for each well) to twice the desired doxycycline concentration, which was progressively varied from 25 to 0 *μ*g/mL. Serial dilutions were then made with LB medium from the first to the 10th column, being the 11th and 12th ones the growth and medium sterility controls, respectively. The cell suspension with density adjusted to 0.5 McFarland was diluted with LB medium and 100 *μ*L were placed in each well. In parallel, purity of the inoculum was checked by incubating aliquots of the bacterial suspension in agar together with the test. Microplates were then incubated at 37°C for 24 h and analyzed visually. MIC was considered to be the doxycycline concentration in the last well that was not clouded.

### 2.4. Cytotoxicity Tests

Citotoxicity tests were done according to the colorimetric ISO 10993-5:2009 assay, Annex C [19]. Because of its ability to react exclusively with active mitochondria, 3-(4,5-dimethylthiazol-2-yl)-2,5-diphenyltetrazolium bromide (MTT) was used to highlight cell viability.

Cultures of the mouse fibroblast cell line (L929, ATCC CCL-1) used as cell culture system were replicated every 48 h, transferred to 75 mL-disposable bottles, and cultivated in the low glucose DMEM containing 10% fetal bovine serum (FBS) in a humidified chamber at 37°C, 5% CO_2_ for 24 h.

Cell cultures for these tests were maintained until the second replicate. Then, the culture medium was discarded and the adhered cells were treated with 0.5% trypsin/0.2% EDTA solution for 15 min at 37°C, taking off the cell monolayer. DMEM plus 10% FBS was added to the resulting cell suspension to neutralize trypsin. Viable cells were counted in a Neubauer chamber through a microscope, model BX 60 Upright Research (Olympus, Center Valley, PA, USA), after staining with 0.4% trypan blue. After adjustment of its concentration to 10^5^ cells/mL with DMEM, the cell suspension was distributed in 96-well microplates (100 *μ*L/well). After incubation of the microplates for cell adherence for 24 h at 37°C, 5% CO_2_, the medium was removed and 100 *μ*L of it was added into wells for both controls (only culture medium = 100% viability; 20% ethanol = 0% viability) and tests (medium containing doxycycline at different concentrations). The plates for tests were again incubated under the same conditions for further checking of cell viability.

After this treatment, the culture medium was discarded from the wells and the MTT solution (1 mg/mL of type I purified water) was added to each well, followed by further incubation at 37°C for 2 h. Then, the MTT solution was discarded and 100 *μ*L of isopropanol was added to each well to dissolve the crystals formed in the mitochondria. The relative cell viability was quantified as a percentage decrease in the absorbance at 570 nm, taking the growth control with culture medium as 100%.

A portion of the effluent was 10-fold concentrated using a vacuum concentrator, model Plus (Eppendorf, Hamburg, Germany), in order to simulate the cytotoxic effect of more concentrated effluents.

## 3. Results and Discussion

### 3.1. Evaluation of Minimum Inhibitory Concentration (MIC) of Doxycycline Effluent from the Fenton Process

Minimum inhibitory concentration (MIC) is the lowest concentration at which a specific antibiotic exerts its inhibitory action against a given microorganism. According to FDA, microorganisms usually utilized in doxycycline tests are* Escherichia coli* ATCC 25922,* Staphylococcus aureus* ATCC 29213,* Enterococcus faecalis* ATCC 29212, and* Pseudomonas aeruginosa* ATCC 27853, for which MIC values lay in the ranges 1.0–4.0, 0.25–1.0, 8–32, and 8–32 *μ*g/mL, respectively [20].

The results of triplicate tests for MIC determination, employing* E. coli* as a target microorganism, are listed in Table 1. Samples obtained from doxycycline dilutions from 6 to 11 exhibited turbidity after 24 hours in all the three experiments, indicating microbial growth.

These results show that pure doxycycline inhibited cell growth up to the fifth-dilution, corresponding to a MIC value of 1.56 *μ*g/mL, which is consistent with the above range reported in literature for* E. coli* [20]. On the other hand, the treated effluent showed no inhibition of microbial growth up to the third-dilution, corresponding to a concentration of 6.25 *μ*g/mL, that is, 25% of that of the initial solution. The initial concentration of the effluent was not further increased with the purpose of avoiding an excess dilution of the culture medium, which would have unavoidably interfered with the kinetics of bacterial growth because of lack of nutrients. This result let us infer that the effluent coming from the Fenton process was not toxic to the microorganism, because no inhibition of cell growth took place under all the dilutions tested. The absence of any inhibition also suggests that the most concentrated effluent sample contained a doxycycline concentration lower than the MIC, which is a proof of the effectiveness of the Fenton process in the treatment of antibiotic-containing effluents.

### 3.2. Evaluation of Cytotoxicity of Doxycycline Effluent from the Fenton Process

**Figure 1:**
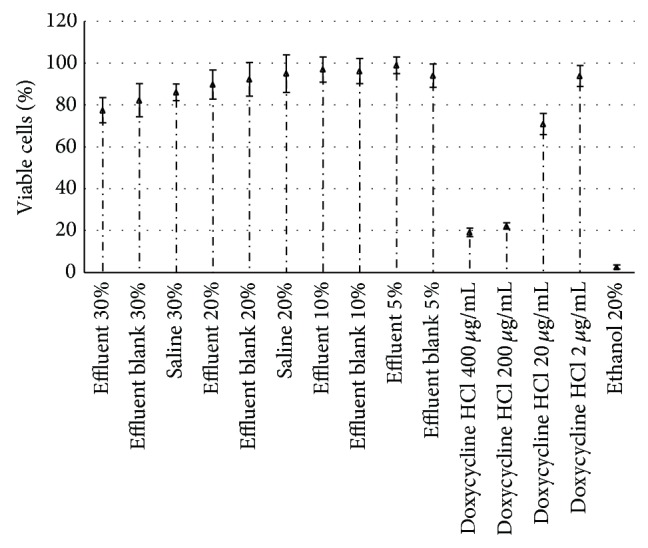
Results of cytotoxicity tests performed by adding the effluent from the Fenton process or doxycycline HCl to the culture medium after 24 h of incubation with L929 cells. Effluent blank did not contain the antibiotic. Ethanol 20% is considered to be responsible for 100% of cell death. Saline solutions are assumed to be inert for cells.

**Figure 2:**
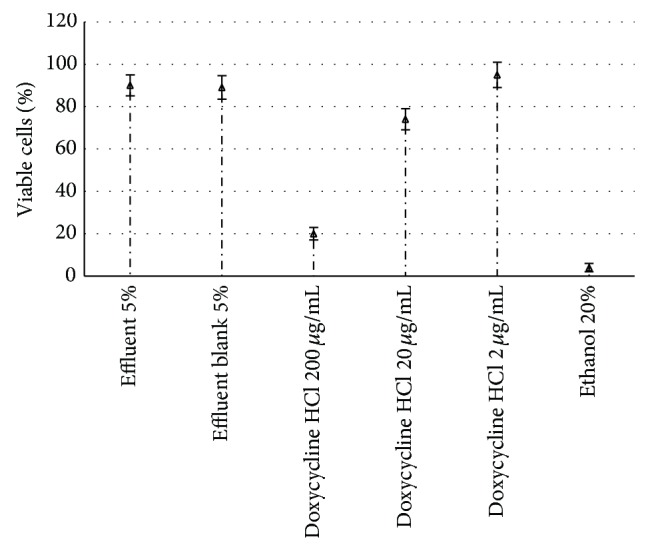
Results of cytotoxicity tests performed by adding the 10-fold concentrated effluent from the Fenton process or doxycycline HCl to the culture medium after 24 h of incubation with L929 cells. Effluent blank did not contain the antibiotic. Ethanol 20% is considered to be responsible for 100% of cell death.

Cytotoxicity tests were carried out in triplicate either on the actual effluent or on a 10-fold concentrated sample obtained by rotary vacuum concentration to simulate the cytotoxic effect of more polluted effluents. The results of these tests are illustrated in Figure 1 using the actual effluent and in Figure 2 using the 10-fold concentrated sample, respectively, where percentages and concentrations utilized to identify the samples do refer to proportions of any product added to cell culture.

As shown in Figure 1, the use of 20 to 30% effluent samples led to a reduction of relative cell viability ranging only from 1 to 22% with respect to that observed using the sole culture medium (100%). However, since no significant difference between the effluent and the blank was observed, one can infer that no subproduct generated by the Fenton process was toxic to L929 cells. On the other hand, doxycycline samples evidenced a clear dose-dependent toxicity of such an antibiotic, being able to reduce cell viability up to 89% at the highest concentration (400 *μ*g/mL).

No significant difference can be observed in Figure 2 between the 10-fold concentrated effluent and the blank. In addition, the further reduction of cell viability compared to the nonconcentrated effluent was so low (from 99 to 90%) as to raise the suspicion that the above effects were likely related to culture medium dilution rather than to an actual toxicity of the effluent components.

## 4. Conclusion

The evaluation of cytotoxicity against L929 cells and MIC of a doxycycline-containing effluent treated by the Fenton process showed that such a compound lost almost entirely its antibiotic characteristics. These results suggest that this process could be successfully exploited in the treatment of industrial effluents containing other antibiotics or in similar environmental applications. The next effort will deal with the characterization of this effluent both before and after the treatment in terms of byproduct identification and quantification.

## Figures and Tables

**Table 1:** Scheme of doxyxycline microdilutions in 96-well microplate for MIC determination using *E. coli* as a target microorganism.

Dilution	1	2	3	4	5	6	7	8	9	10	11	12
*C*_Doxycycline_ (*µ*g/mL)	25.0	12.5	6.25	3.12	1.56	0.78	0.39	0.19	0.10	0.01	0	0
Turbidity	−	−	−	−	−	+	+	+	+	+	+	+
